# Occurrence of Thermotolerant *Hartmannella vermiformis* and *Naegleria* Spp. in Hot Springs of Ardebil Province, Northwest Iran

**Published:** 2012

**Authors:** R Solgi, M Niyyati, A Haghighi, E Nazemalhosseini Mojarad

**Affiliations:** 1 Department of Medical Parasitology and Mycology, School of Medicine, Shahid Beheshti University of Medical Sciences, Tehran, Iran; 2 Research Center for Gastroenterology and Liver Diseases, Shahid Beheshti University of Medical Sciences, Tehran, Iran

**Keywords:** Hot springs, *Hartmannella*, *Naegleria*, Iran

## Abstract

**Background:**

Geothermal waters could be suitable niches for thermophilic free living amoebae including *Naegleria* and *Hartmannella*. Ardebil Province, northwest Iran is popular for having many hot springs for recreational and health purposes activity. The present research is the first molecular based investigation regarding the presence of *Naegleria* and *Hartmannella* in the hot springs of Ardebil Province in Iran.

**Methods:**

Overall, 30 water samples were taken from waters of thermal hot springs in Ardebil Province, Iran during 2010-2011. All collected samples were transferred to Dept. of Parasitology and Mycology, Shahid Beheshti University of Medical Sciences, Tehran, Iran. Cultivation of concentrated water samples was performed using culture-enrichment method. Cloning of the target amoebae was obtained and morphological and molecular analysis was done using page key combined with two sets of primers, respectively. Sequence analysis and homology search was used for strains identification.

**Results:**

Of 30 water samples, 8 (26.7%) were positive for thermotolerant Vahlkampfiids and *Hartmannella* based on morphological characteristics of vegetative form and double walled cysts. Cloning of the target amoebae were done successfully. Sequencing of the positive isolates revealed that the strains belonged to *Naegleria* (*N. carteri* and *N. spp*) and *H*. *vermiformis*.

**Conclusion:**

The result highlights a need for improved filtration and disinfection and periodic monitoring of recreational thermal waters in order to prevent disease related to free- living amoebae. This is the first comprehensive molecular study of thermophilic *Naegleria* and *Hartmannella* in hot springs of Iran.

## Introduction

Free-living amoebae (FLA) including various families which some of them are classified as potentially pathogenic organisms for human and animals (1, 2). The family Vahlkampfiidae has been found in environmental sources such as fresh water, soil, dust and clays (3). To date, the genera of *Naegleria*, *Vahlkampfia* and *Paravahlkampfia* introduced as potentially pathogenic amoebae for human (4–6). Indeed, recent report regarding pathogenic potential of *Vahlkampfia in* Iran leads to more attention regarding this free-living organism (4). The genus *Naegleria* spp. consists of 30 different species, two of which (*N. fowleri* and *N. australiensis)* have been described as potential pathogenic organisms and they are the causative agents of fulminant meningoencephalitis (1). These amoebae are able to tolerate extremes of temperature and thus thermal waters could be an ideal environment for *Naegleria* growth and survival (1, 3). It should be mentioned that nonpathogenic *Naegleria* could also be an important threat for human, since these avirulent amoebae could harbor pathogenic microbes and could act as Trojan horse (7). A previous research in Iran revealed the presence of vahlkampfiids in thermal waters of Sarein City (8), however this reported study were based on only morphological criteria. It is important to mention that morphological criteria can reveal the presence of vahlkampfiids, however, molecular analysis is necessary for genera identification of Vahlkampfidae family (9).

The family Hartmannellidae also includes thermotolerant amoebae such as *Hartmannella vermiformis*. There are recent reports regarding the pathogenic potential of this free living organism (10). A case of mixed keratitis infection has been reported due to *Acanthamoeba* and *H*. *vermiformis* (10).

Ardebil Province in the northwest of Iran is a famous place for having many hot springs, hot tubs, spas, and mineral waters. These thermal waters have been used for recreational and health purpose. High temperature of thermal springs is suitable factor for growth of thermotolerant amoebae such as *Naegleria* and *Hartmannella* (1). Indeed, the ability of these ubiquitous organisms to tolerate high temperatures made thermal waters favorable niches.

The present research is the first molecular based investigation regarding the presence of *Naegleria* and *Hartmannella* in hotsprings of Ardebil Province in Iran. The occurrence of these amoebae highlights the need of more monitoring of such waters and reflects periodic surveillance of recreational hot springs in Iran.

## Material and Methods

### Sampling

Overall, 30 water samples were taken from thermal hot springs in Ardebil Province, Iran during 2010-2011. All of the cities which contained recreational hot springs have included in the present study such as Sarein, Meshkin shahr, Nir, Ardebil and Givi. Briefly, 500 ml of surface water were collected from hot springs and transferred to the Department of Parasitology and Mycology, School of Medicine, Shahid Beheshti University of Medical Sciences, Iran. All hot springs included were used for both recreation and for health purposes.

### Filtration, cultivation and cloning

Samples were filtered through cellulose nitrate membranes with a pore size of 1.2 µm. The filters were then inverted and transferred onto 1% non-nutrient agar plates covered with a thin layer of autoclaved *Escherichia coli*. Positive plates were screened for vahlkampfiids and *Hartmannella* spp. using both optical and inverted microscopes based on pages key (11). Cloning of the candidate amoebae were performed using culture replicates according to our previous study (12).

### PCR amplification and gel electrophoresis

Amoebae were harvested from plates and washed using phosphate-buffered saline (PBS pH 7). DNA Extraction was performed using the Instagene matrix (Chelex; Biorad). Briefly, approximately 1000 cells were incubated with 50 µl Chelex. Incubation was done at 56°C for 20 min. Additional incubation were performed for 10 min using boiling water. DNA pellet was obtained by centrifuging the samples at 10 000 g for 5 min and the supernatant was used as the DNA template for PCR reaction. Modified phenol-chloroform methods were performed for DNA extraction of cysts according to our previous study (12).

The PCR reaction was performed in 30 µl Ampliqone (Taq DNA Polymerase Master Mix Red, Denmark) as a readymade mixture. Briefly, 25 µl of master mix with 5 ng DNA templates and 20 pmol primers were combined to achieve a volume of 30 µl. Two sets of primers were used for identification of vahlkampfiids and *Hartmannella* spp. The first set was ITS primers which were able to detect *Naegleria* spp. and they are designed to obtain a 400-430 bp PCR product (13). The sequences of ITS primers were: forward 5'GAACCTGCGTAGGGATCATTT 3’ and reverse primer ITS2 5’ TTTCTTTTCCTCCCCTTATTA 3’. The second set was a primers which could amplify a fragment of 18s rRNA gene of *Hartmannella* (12, 14). The sequences were: forward 5'GCT CCA ATA GCG TAT ATT AA 3’ and revers 5’ AGA AAG AGC TAT CAA TCT GT 3’. Each PCR cycling condition included 35 cycles of denaturation at 94°C for 1 min, followed by 35 repetition cycles at 94°C for 35 s, annealing at 56°C for 45 s, and extension at 72°C for 1 min.

Gel electrophoresis were performed to detect PCR products using 1.5% agarose gel stained with a solution of ethidium bromide (25 mg ml-1) and examined under UV illumination.

### Sequencing of the PCR products

PCR-products were submitted to sequencing using an ABI 3130X automatic sequencer at the Research Center for Gastroenterology and Liver Diseases, Shahid Beheshti University of Medical Sciences, Tehran. Homolgy analysis of the obtained sequences with genes in the gene data bank was done using BLAST software from the National Center for Biotechnology Information (NCBI) site. The highest homology and query overage was the base of strains identification.

## Results

**Fig. 1:**
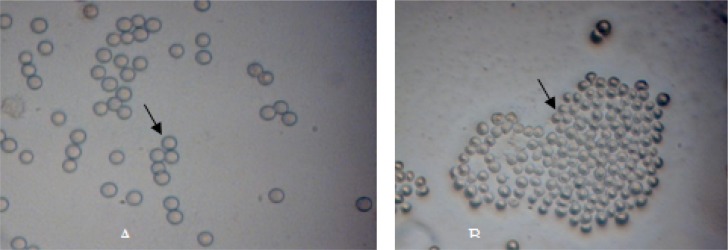
(A) Light micrograph of *Naegleria* cysts in non-nutrient agar x400 (B) Light micrograph of *Hartmannella vermiformis* cysts in non-nutrient agar x400

Overall, out of 30 water samples, 8 (26.7%) were positive for vahlkampfiids and Hartmannellidae family based on morphology characteristics. Temperatures and pH of thermal springs were ranged from 41-53° C and 4.90-7, respectively (Table 1). Vahlkmapfiids identification was based on spherical double wall cysts and temporarily branched trophozoites (Fig. 1). *Hartmannella* were also characterized using morphological criteria including small spherical or ovoid cyst shape and outer wall of some were separated (Fig. 1). The trophozoite form was limax containing one small nucleolus. Cloning of the candidate amoebae were done successfully after 2 months.

**Table 1:** Location and distribution of Free- living *Naegleria and Hartmannella* in hot springs of Ardebil province, Iran

Code	Locality	Water type	pH	Tem (°C)	Genus	Accession number
HSS1	Sarein	bicarbonate spring	6.15	46	*Naegleria (N. carteri)*	**JQ023595**
HSS5	Sarein	bicarbonate spring	6.28	41	*Hartmannella vermiformis*	**JQ023592**
HSS9	Sarein	bicarbonate spring	7.00	42.0	*Hartmannella vermiformis*	**JQ023591**
HSS3	Sarein	bicarbonate spring	6.07	43.50	*Hartmannella vermiformis*	**JQ023590**
HSM1	Meshkin shahr	Sulfur spring	4.90	45.1	*Hartmannella vermiformis*	**JQ023593**
HSM1	Meshkin shahr	Sulfur spring	4.90	45.1	*Naegleria spp*.	**JQ023596**
HSM2	Meshkin shahr	bicarbonate spring	6.39	48.2	*Hartmannella vermiformis*	**JQ023594**
HSN1	Nir	Sodium chloride	6.18	53	*Naegleria spp*.	**JQ023597**

* HSS: Hot springs Sarein/HSM: Hot spring Meshkin shahr/HSN: Hot spring Nir

**Fig. 2:**
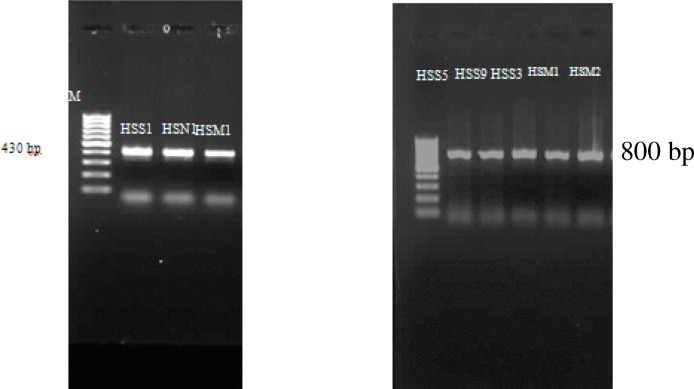
PCR products of the isolated strains (*Hartmannella* spp. and *Naegleria* spp.) from hot springs of Ardebil province, Iran

A 400 bp and 800 bp PCR products were obtained for vahlkampfiids and *Hartmannella*, respectively (Fig. 2). Sequence analysis of the PCR products revealed that the three vahlkampfiid amoebae belonged to the *Naegleria* genera (Isolates: HSS1, HSN1, HSM1). Basic local alignment search tool (BLAST) showed that one of strains had a high homology to *N. carteri* (Accession number: AM167887.1). Moreover, all of the *Hartmannella* isolates detected in the present study were identified as *H. vermiformis*.

Nucleotide sequence accession numbers were deposited in the GenBank database (accession number: **JQ023590-JQ023597**).

## Discussion

The present study is the first molecular identification of FLA belonging to the *Naegleria* spp. and *H. vermiformis* in hot springs of Iran. Presence of potentially pathogenic FLA could be a serious hazard for people using such waters. It is important to mention that some of thermal waters in this region are used for recovery of eye trauma. Therefore, the occurrence of *H. vermiformis* in thermal waters could lead to the exposure of high risk people to thermotolerant amoebae. Recent reports reflect the pathogenic potential of *H. vermiformis* for human cornea (10, 15). This is in agreement with Lorenzo et al. (2007) study who revealed the presence of mixed keratitis infection due to *Acanthamoeba* and *Hartmannella* (10). *Hartmannella* amoebae are also considered as suitable hosts for pathogenic microorganisms including *Legionella pneumophila* and *Pseudomonas* (16). Previous researches stated that Hartmannellid amoebae are an important growth factor for *L. pneumophila* (16). Indeed, various factors including the ability of *Hartmannella* to grow at temperatures above 40 ^0^C and the isolation of this thermotolerant amoebae from amoebic keratitis patients emphasize that *Hartmannella* could be a potential pathogen for human (10). Moreover, according to previous researches it has been found that *Hartmannella* isolated from keratitis patient could lead to cytotoxicity on epithelial corneal cells (10). To this end, presence of *Hartmannella* in hot springs should be considered as health hazard.

The present study also reports the occurrence of *Naegleria* sp. based on molecular approaches. This is the first report of *N. carteri* in Iran. It is important to note that although the identified *Naegleria* are non-pathogenic and they have not isolated from clinical cases yet, but they could be a suitable host for pathogenic microorganisms (17). A previous research reported that *N. pagei* could coexict with pathogenic *L. pneumophila* (17). Indeed, non-pathogenic free living amoebae must consider as a carrier of pathogenic microbes (18). It should be noted that in one springs we have identified mixed (isolates: HSM1) amoebae belonging to *Hartmannella* and *Naegleria*. Filtration of this contaminated spring was not adequate for decontamination of water.

In conclusion, the result of the present study highlights an urgent need for improved filtration and disinfection and periodic monitoring of thermal waters in order to prevent disease related to thermotolerant free living amoebae.
